# Next Generation Mapping of Enological Traits in an F_2_ Interspecific Grapevine Hybrid Family

**DOI:** 10.1371/journal.pone.0149560

**Published:** 2016-03-14

**Authors:** Shanshan Yang, Jonathan Fresnedo-Ramírez, Qi Sun, David C. Manns, Gavin L. Sacks, Anna Katharine Mansfield, James J. Luby, Jason P. Londo, Bruce I. Reisch, Lance E. Cadle-Davidson, Anne Y. Fennell

**Affiliations:** 1 Horticulture Section, School of Integrative Plant Science, Cornell University, Geneva, New York, United States of America; 2 Bioinformatics Facility, Institute of Biotechnology, Cornell University, Rhodes Hall, Ithaca, New York, United States of America; 3 Department of Food Science, Cornell University—NYSAES, Geneva, New York, United States of America; 4 Department of Food Science, Cornell University, Ithaca, New York, United States of America; 5 Department of Horticultural Science, University of Minnesota, St. Paul, Minnesota, United States of America; 6 USDA-ARS Grape Genetics Research Unit, Geneva, New York, United States of America; 7 Plant Science Department, South Dakota State University, Brookings, South Dakota, United States of America; 8 BioSNTR, Brookings, South Dakota, United States of America; Wuhan Botanical Garden of Chinese Academy of Sciences, CHINA

## Abstract

In winegrapes (*Vitis* spp.), fruit quality traits such as berry color, total soluble solids content (SS), malic acid content (MA), and yeast assimilable nitrogen (YAN) affect fermentation or wine quality, and are important traits in selecting new hybrid winegrape cultivars. Given the high genetic diversity and heterozygosity of *Vitis* species and their tendency to exhibit inbreeding depression, linkage map construction and quantitative trait locus (QTL) mapping has relied on F_1_ families with the use of simple sequence repeat (SSR) and other markers. This study presents the construction of a genetic map by single nucleotide polymorphisms identified through genotyping-by-sequencing (GBS) technology in an F_2_ mapping family of 424 progeny derived from a cross between the wild species *V*. *riparia* Michx. and the interspecific hybrid winegrape cultivar, ‘Seyval’. The resulting map has 1449 markers spanning 2424 cM in genetic length across 19 linkage groups, covering 95% of the genome with an average distance between markers of 1.67 cM. Compared to an SSR map previously developed for this F_2_ family, these results represent an improved map covering a greater portion of the genome with higher marker density. The accuracy of the map was validated using the well-studied trait berry color. QTL affecting YAN, MA and SS related traits were detected. A joint MA and SS QTL spans a region with candidate genes involved in the malate metabolism pathway. We present an analytical pipeline for calling intercross GBS markers and a high-density linkage map for a large F_2_ family of the highly heterozygous *Vitis* genus. This study serves as a model for further genetic investigations of the molecular basis of additional unique characters of North American hybrid wine cultivars and to enhance the breeding process by marker-assisted selection. The GBS protocols for identifying intercross markers developed in this study can be adapted for other heterozygous species.

## Introduction

The inherent role of the enological properties of a winegrape cultivar is undeniable in the final taste of wine. While both growing conditions and choice of vinification procedures influence eventual wine properties [1–4], the grapevine cultivar has arguably the largest effect on wine quality [5, 6]. Understanding the genetic controls of enologically important traits will assist breeders, viticulturists, and enologists to delineate strategies for improving wine quality.

Grapevine is a cross pollinating, highly heterozygous crop that exhibits inbreeding depression in the form of diminished seed viability and reduced vine vigor. Because of this, grapevine breeders typically employ breeding schemes based on background selection for *V*. *vinifera* fruit quality and foreground selection for introgressed traits [7, 8], which are applied under a framework of modified-backcrossing [2, 8].

Winegrape breeding began in earnest in the 19^th^ C., as breeders attempted to develop grapevines with improved pest and disease resistance by cross-hybridizing popular *V*. *vinifera* winegrape cultivars with resistant North American wild species such as *V*. *aestivalis* Michx, *V*. *cinerea* (Engelm. ex A. Gray) Engelm. ex Millard, *V*. *labrusca* L., *V*. *riparia* Michx and *V*. *rupestris* Scheele [9].While these introgressions have positive adaptive traits including disease resistance, pest resistance, and cold hardiness [5, 10, 11], they often have inferior fruit composition for winemaking [9, 12].

Common quality parameters for wine grapes include titratable acidity (TA), soluble solids (SS) content as a proxy for sugar concentration, and yeast assimilable nitrogen (YAN) [13]. Sugars increase during berry ripening, and an SS of 20–24% w/w (Brix) is typically targeted for production of a dry table wine with 11–14% v/v alcohol. Conversely, malic acid (MA)–one of the two major grape organic acids that contribute to TA–is respired during berry ripening [14]. While SS contents of wild *Vitis* and *V*. *vinifera* are comparable, MA concentrations in wild *Vitis* are generally much higher–often in excess of 10 g/L at harvest [15]–and wines produced from *Vitis* interspecific hybrids are frequently reported to have excessive acidity and sourness [5] if not ameliorated before bottling. This situation is exacerbated in cooler climates where hybrid grapes are more widely used, such as the northern United States, because of slower MA respiration [16]. Thus, a key goal following trait introgression is combining lower MA content with appropriate sugar content. YAN is also widely measured in winegrapes prior to fermentation, since insufficient concentrations (< 150 mg/L) can lead to stuck fermentations and off-odor formation [17], while excessive concentrations can lead to formation of ethyl carbamate, a known carcinogen [1]. YAN concentrations at both extremes have been reported in interspecific hybrids [18], and breeding cultivars with appropriate YAN content is desirable.

Genetic controls of these key compositional factors have only recently been investigated in grapevine [19]. The formation of MA in the grape berry involves the glycolysis and citric acid (TCA) cycle and is regulated by the malate metabolism pathway. The diverse pathways in metabolic processes and environmental factors, such as temperature during ripening, all contribute to the complexity for dissecting the molecular basis of MA content. Two recent studies reported several QTLs controlling total SS and acid content [20, 21]. However, the identified QTLs could only explain a small percentage of the total phenotypic variance (< 17%), and candidate genes could not be identified. QTL analysis of YAN in grape berries has not been attempted, even though YAN is known to vary considerably among both *V*. *vinifera* [22] and *Vitis* spp. hybrids [18].

Given the heterozygosity and inbreeding depression in *Vitis*, QTL mapping studies generally use F_1_ full-sib progenies and the pseudo-testcross method [23], with 70 to 300 progeny and from 100 up to 1,826 molecular markers [20, 24–27]. Previously, a family of 119 F_2_ progeny was developed from selfing an F_1_ individual derived from the cross *V*. *riparia* × *Vitis* hybrid ‘Seyval’ [28], and a linkage map based on 120 simple sequence repeats (SSRs) was developed [29]. ‘Seyval’ is a complex interspecific hybrid of *V*. *vinifera* (55% by pedigree) with wild species native to the south-central United States and is a relatively cold hardy white wine cultivar, resistant to disease and the phylloxera aphid *Daktulosphaira vitifoliae* (Fitch 1855). *V*. *riparia* has been used to breed for early acclimation and freezing tolerance in hybrid winegrapes [28]. The acclimation and dormancy characteristics of *V*. *riparia* and ‘Seyval’ have been studied at the physiological, transcriptomic and metabolomic levels providing additional gene level characterization [28–32].

Genotyping by sequencing (GBS) is a marker platform that uses restriction enzymes to reduce genome complexity prior to next generation sequencing. GBS has become widely used due to several benefits: simultaneous marker discovery and genotyping, high sample throughput and scalability, and high resolution at a low per-marker cost [33]. However, due to arbitrary sampling of sites and the high level of multiplexing typical in GBS, sequencing depth is usually reduced, leading to missing data and heterozygote undercalling, in which heterozygous sites are undersampled and wrongly interpreted as homozygous [34]. While heterozygote undercalling has been irrelevant to GBS applications in inbred species, it is a critical problem in grapevine and other highly heterozygous and diverse species, requiring the development of new methodologies.

The present study demonstrates the use of novel GBS analysis methods to develop an improved linkage map for the F_2_ mapping family derived from *V*. *riparia* × ‘Seyval’ [35]. This GBS map was validated by analysis of the berry skin color locus, previously characterized [36–38]. Our results indicate this is an effective approach for mapping QTL and identifying candidate genes in *Vitis*, including preliminary results for SS, MA and YAN.

## Materials and Methods

### Plant material

The F_2_ mapping family was generated as described in Fennell et al. [28]. The F_2_ progeny were derived from self-pollination of a single hermaphrodite F_1_ individual (16-9-2) generated from a cross of *V*. *riparia* (USDA PI 588259) × ‘Seyval’ (Seyve-Villard 5–276). One hundred nineteen F_2_ seedlings were germinated from seed, grown in the greenhouse, cycled into dormancy and cuttings taken for vegetative propagation. After chilling fulfillment, cuttings were rooted and vines grown for field establishment as well as maintenance in a controlled environment. Ecodormant one-year-old vines were planted in the field in spring 2005, trained and maintained for fruiting. A first genetic map for this family was published in 2009 using SSR markers [29]. Fruit from the F_1_ parent, the *V*. *riparia* grandparent, and 65 fruiting progeny with greater than 20 clusters were harvested 30 days post veraison, and 150 g of randomly collected berries were flash frozen and sent to Cornell University for berry quality phenotyping in 2013. Fruit ripening parameters particularly SS and acid content are impacted by changes in temperature. This population had ripening times spanning early to late season; therefore, harvesting at 30 days post veraison avoided low night temperature exposure that would have impacted the late season genotypes. Additional F_2_ seeds were generated in 2011 and 2012. Three hundred fourteen additional seedlings were germinated in 2013 and genotyped for increased map resolution.

### Phenotyping

Frozen berry samples (150 g) from each individual were destemmed, allowed to partially thaw, and homogenized in a 250-mL stainless steel Waring blender (Waring laboratory science, Stamford, CT, USA) at medium speed for 30 s followed by high speed for 30 s. A 10 g portion of berry slurry was transferred to a 15-mL plastic centrifuge tube and then frozen at -20°C. Prior to analysis, samples were thawed. Juice soluble solids (Brix) were analyzed by refractometer (Misco Model #PA203X; Misco, Cleveland, OH USA). The MA was measured on an Agilent 1260 infinity series HPLC using a previously reported method [39]. The YAN was measured as the sum of ammonium and primary amino nitrogen using commercial enzymatic assays (Unitech Scientific, LLC., Hawaiian Gardens, CA, USA) on a ChemWell model 2900 analyzer (Awareness Technologies, Palm City, FL, USA). A subset of MA analyses (one out of every ten individuals) were subjected to either injection replication or extraction replication. The relative standard deviation (%RSD) for MA analyses was 0.4% and 0.7% for injection alone or extraction/injection, respectively. A set of standards and blanks was run every ten samples to verify the retention time and response factor of analytes being measured. An analogous approach was used for replication of YAN analyses. Technical replicates of SS measurements were not conducted due to the high reproducibility of the method.

### Genotyping

For each vine, a single small leaf (less than 1 cm diameter) was placed in a tube of a Costar 96-well cluster tube collection plate (Corning Life Sciences, Tewksbury, MA, USA). Each 96-well plate consisted of 91 genotype-specific samples, two sets of duplicates and one blank well. The location of the blank well in each plate was unique as a plate identity control. Leaf tissues were maintained at 4°C during and after harvest. In the laboratory, two stainless steel genogrinder beads were placed in each tube and plates were frozen at -80°C. Tissue grinding took place in a Geno/Grinder 2000 (OPS Diagnostics LLC, Lebanon NJ, USA) with 96-well plates agitated in pairs at 400X speed for 1 minute. Plates were then stored at -80°C until processing with DNeasy 96-well DNA extraction kits (Qiagen, Valencia CA, USA). Modifications were made to the manufacturer’s protocol to improve DNA quality and quantity as follows: 1) PVP-40 (2% w/v) was added to the AP1 lysis buffer prior to heating; and 2) visual inspection for complete re-suspension of the sample pellet of each 8-tube strip was added to the agitation step following AP1 addition.

Genotyping-by-sequencing (GBS) was performed as described by Elshire et al. (2011) [35], integrating four 96-well plates across 384 barcodes for library preparation and sequencing. For SNP calling, the raw sequence data for the 424 F_2_ progeny plus the F_1_ progenitor (16-9-2) was processed through the TASSEL 3.0 GBS pipeline [33] using the 12X.v2 *V*. *vinifera* ‘PN40024’ reference genome [40] from The French-Italian Public Consortium (https://urgi.versailles.inra.fr/Species/Vitis/Data-Sequences/Genome-sequences) for alignment and the Burrows–Wheeler Aligner (BWA) mem [41] with default parameters. The output consisted of variant call format (VCF) file version 4.1 [42] including SNPs present in at least 40% of the progeny and with a minor allele frequency (MAF) ≥ 0.1. Subsequently, the VCF was filtered using vcftools ver. 1.12a [42] and TASSEL [43] versions 3.0 and 4.0. A total of 291,453 SNPs were identified in 424 F_2_ progeny by TASSEL 3.0, then a custom filtering process was applied for alignment. The filtering was based on keeping sites with a minimum read depth of 6 and 75% completeness by site across progeny and by progeny across sites. Results were output as a TASSEL hapmap file. Finally, using a custom perl script (S1 File) markers heterozygous in the F_1_ progenitor and with a co-dominant 1:2:1 segregation among the F_2_ progeny were identified by a chi-squared (χ^2^) goodness-of-fit test at α≤0.01. These were reformatted to be imported in JoinMap® 4.1.

### Map construction

For map construction, the intercross markers described above were coded as *hk×hk* (assumption of no dominance because the gametic phases were unknown) to be imported in JoinMap4® 4.1 Build: 31jul13.4feb11. Cross-pollination (CP) cross type was used to estimate linkages [44]. This type of marker segregation is particularly useful when performing linkage mapping in outbreeding species because the markers are symmetrical with respect to parental segregation and because they allow construction of integrated linkage maps even when the identification of tri or tetra-allelic markers is insufficient [45]. For the present study, these markers, in conjunction with the multipoint maximum likelihood algorithm implemented in JoinMap^®^ [46], allowed linkage calculation and phasing determination, which enabled us to recode marker genotypes based on predicted phase, and then perform QTL analyses as in an F_2_ experimental cross.

Prior to grouping and ordering, markers with highly significant (*p≤0*.*00001*) deviation from Mendelian expectations and loci duplicating genetic information were filtered in JoinMap^®^. For grouping, a linkage-independence LOD ≥10 was used. For ordering and genetic location determination, the Maximum Likelihood mapping algorithm and Kosambi mapping function were applied, in addition to three rounds of map optimization. Given the amount of missing data allowed, genetic distance inflation occurred among markers. Thus, to keep the most reliable markers and their locations while limiting distance inflation, a threshold of 2.5 cM for the nearest neighbor stress (N.N. stress) was considered. This is an empirical threshold determined through the linkage mapping of 16 full-sib families analyzed in the USDA-NIFA Specialty Crops Research Initiative *Vitis*Gen project (www.vitisgen.org).

Sixty-five F_2_ progeny were included for evaluation of berry skin color, for which black (pigmented) and white (nonpigmented) were recoded as 1 and 0, respectively. QTL analysis was performed using the standard interval mapping procedure (EM algorithm) with binary model in the package R/qtl [47] version 1.36.5 for R ver. 3.1.2 [48] and using F_2_ as the cross type. One thousand permutations at α  = 0.05 were executed to calculate the LOD threshold. The additive and dominance QTL effects were estimated taking missing genotype information into account. Missing genotypes were simulated given observed marker data by the hidden Markov model (sim.geno function in R/qtl). Reports were generated for maximum LOD score, 1.5-LOD support interval in cM, and the physical location in the reference genome in Mbp, as well as the percentage of variation explained (R^2^).

The QTL mapping of the quantitative traits SS, MA and YAN was performed in two stages: 1) for each trait independently using composite interval mapping through the package R/qtl [47] version 1.36.5 for R ver. 3.1.2 [48]; and 2) joint QTL mapping for multi-trait trials in a single environment, using the routines QMTQTLSCAN, QMTESTIMATE and QMTBACKSELECT [49] integrated in the statistical package GenStat for Windows 17^th^ [50].

For the three traits, phenotypic information was used from fruit of 63 progeny harvested in 2013. The normality of the distributions was tested by the Shapiro-Wilks tests. Logarithm (Log) transformation was applied for results validation. The QTL detected were similar using either transformed or original data. The markers were coded according to the recommendations of the software used (A, B and H for R/qtl, and as 1|1, 2|2 and 1|2 in GenStat). QTL mapping was executed as described for the berry skin color in R/qtl. The 1.8-LOD support intervals were reported as suggested by “A Guide to QTL Mapping” [51] for mapping traits for an intercross family.

In the case of the joint QTL mapping in GenStat, the joint QTL mapping was performed using the phenotypic data for each combination of two traits (MA-SS, MA-YAN and SS-YAN). Cofactors were placed every 1.51 cM along the genome, resulting in placement of 861 cofactors along the genetic map. The model used for the first QTL scan was ${y_{ij}=\mu +T_{j}+\sum_{f\in F}\left[\left(x_{if}^{add}c_{jf}^{add}+x_{if}^{dom}c_{jf}^{dom}\right)+\left(x_{i}^{add}a_{j}^{add}+x_{i}^{dom}a_{j}^{dom}\right)\right]+TE}_{ij}$, where *y*_*ij*_ is the value of trait *j* for genotype *i*, *T*_*j*_ is the trait main effect, *F* is the set of cofactors, *x*_*if*_^*add*^ and *x*_*i*_^*add*^ are the additive genetic predictors of genotype *i* at the cofactor positions and at the tested position, respectively. The associated effects are denoted by *c*_*jf*_^*add*^ and *α*_*j*_^*add*^ for cofactors and tested positions, respectively. *x*_*if*_^*dom*^ and *x*_*i*_^*dom*^ are dominance genetic predictors of genotype *i* at the cofactor positions and at the tested position, respectively, with associated effects *c*_*jf*_^*dom*^, and *α*_*j*_^*dom*^. *TE*_*ij*_ refers to the residual error of the trait *j* for genotype *i*. The fit of the unstructured model was iteratively performed through Restricted Maximum Likelihood (REML), using 1000 iterations. The QTL threshold was set at a significance level of 0.05 using the Li and Ji method [52] with minimum cofactor proximity of 50 cM and minimum separation of selected QTLs of 30 cM.

Subsequently for the interval mapping and estimation of QTL effects, the model was simplified to: ${y_{ij}=\mu +T_{j}+\sum_{l\in L}\left[\left(x_{il}^{add}a_{jl}^{add}+x_{il}^{dom}a_{jl}^{dom}\right)\right]+GT}_{ij}$, where *y*_*ij*_ is the value of trait *j* for genotype *i*, *T*_*j*_ is the trait main effect, *x*_*il*_^*add*^ are the additive genetic predictors of genotype *i* for locus *l*, and *α*_*jl*_^*add*^ are the associated effects. *x*_*il*_^*dom*^ are the dominance genetic predictors, and *α*_*jl*_^*dom*^ are the associated effects. The method to fit these models follow the ideas of Malosetti et al. [53] and Boer et al. [54]. Both mixed models consider the genotypes as random terms, while the genetic predictors as fixed effects. The genetic predictors can be considered as genotypic covariables informing the genotypic composition of a genotype at a specific chromosome locus [55]. *GT*_*ij*_ is assumed to follow a multi-Normal distribution with mean vector 0, and a variance covariance matrix Σ, which was modeled during the QTL mapping procedure.

Finally, to approach the causality between traits MA and SS (causal, reactive, independent, full), a causal model selection test [56] was implemented using the function cmst in the R package qtlhot version 0.9.0 [57].

## Results

### Marker development

**Fig 1:**
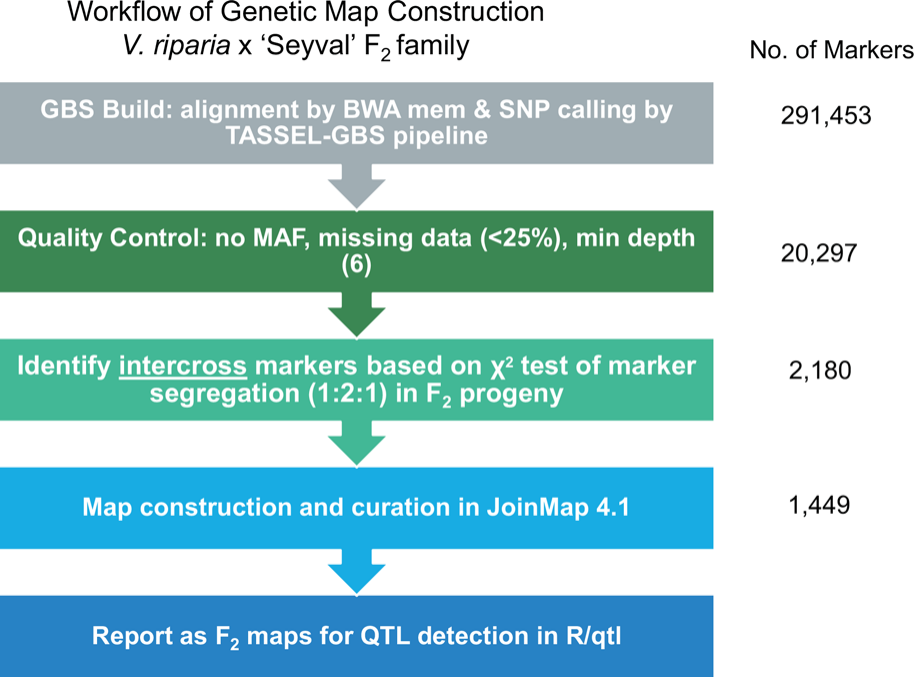
Workflow of marker development and genetic map construction in the *V*. *riparia* × ‘Seyval’ F_2_ family. The left panel shows the five main steps with filtering parameters, and the right panel shows the numbers of markers resulting from each step.

A total of 291,453 quality single nucleotide polymorphisms (SNPs) were identified in 424 F_2_ progeny relative to the *V*. *vinifera* ‘PN40024’ reference genome, version 12X.v2. Subsequently SNP filtering by minimum read depth and missing data rate yielded 2180 intercross markers with 1:2:1 segregation in the progeny, as determined by a χ2 test of marker segregation. The markers were discovered and coded as *hk×hk* using a custom perl script (S1 File). Further marker curation and genetic map construction were performed in JoinMap^®^ 4.1, resulting in a final set of 1,449 markers. The workflow for marker development and linkage map construction in the F_2_ mapping family are presented in Fig 1.

### Linkage map construction

**Fig 2:**
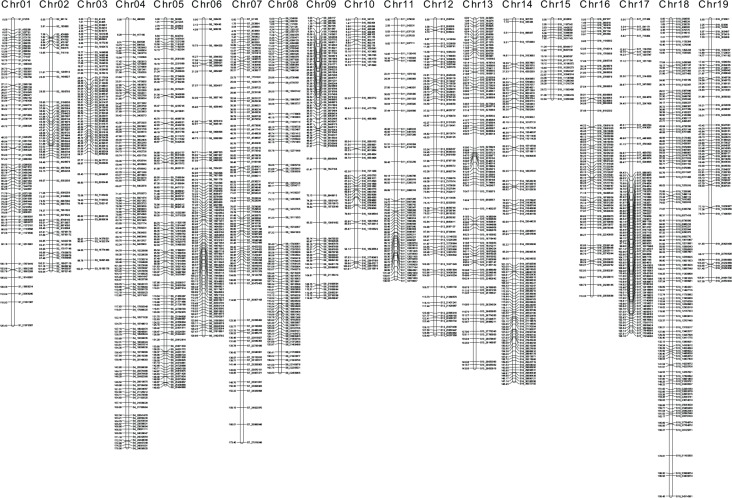
Linkage map of F_2_ mapping family derived from *V*. *riparia* × ‘Seyval’. The linkage map consists of 1,449 markers with a total map size of 2423.9 cM. The 19 chromosomes are labeled at the top of each linkage group. Genetic positions (cM) and physical locations (‘PN40024’ reference version 12X.v2) are listed to the left and right of each chromosome, respectively.

Linkage analysis using LOD 10.0 grouped the 1449 markers into 19 linkage groups (LG) with a total map size of 2423.9 cM and a map density (average distance between markers) of 1.67 cM (Fig 2). Given the grapevine genome size of about 458.8 Mb [40], the current genetic map is about 0.189 Mb/cM. Based on a comparison of the genetic map to the reference genome (physical map), all linkage groups (except Chr15) had an estimated genome coverage greater than 90%, and the average genome coverage was 95.1% (S2 File). To check marker order, the genetic positions of markers within each LG were compared with physical coordinates on the reference genome (S3 File). Marker order was conserved between the genetic and physical map.

Compared to an SSR map previously developed for the same F_2_ family [29], this GBS map represented a nine-fold improvement in resolution from 14.9 cM to 1.67 cM between markers and an 11% increase in the average genome coverage, from 84% to 95% (Table 1). In addition, there were six gaps greater than 20 cM in the previous SSR map, while the largest gap in the GBS map was 13.5 cM.

**Table 1:** Comparison between the previously published SSR-based map and the GBS-based map.

	SSR map	GBS map
No. of progeny	119	424
No. of markers	120	1449
No. of linkage groups	21	19
Map size (cM)	1784	2424
Average distance between markers (cM)	14.87	1.67
Average genome size per cM (Mb/cM)	0.257	0.189
Genome coverage (%)	83.7	95.1

### Berry color mapping for map validation

**Fig 3:**
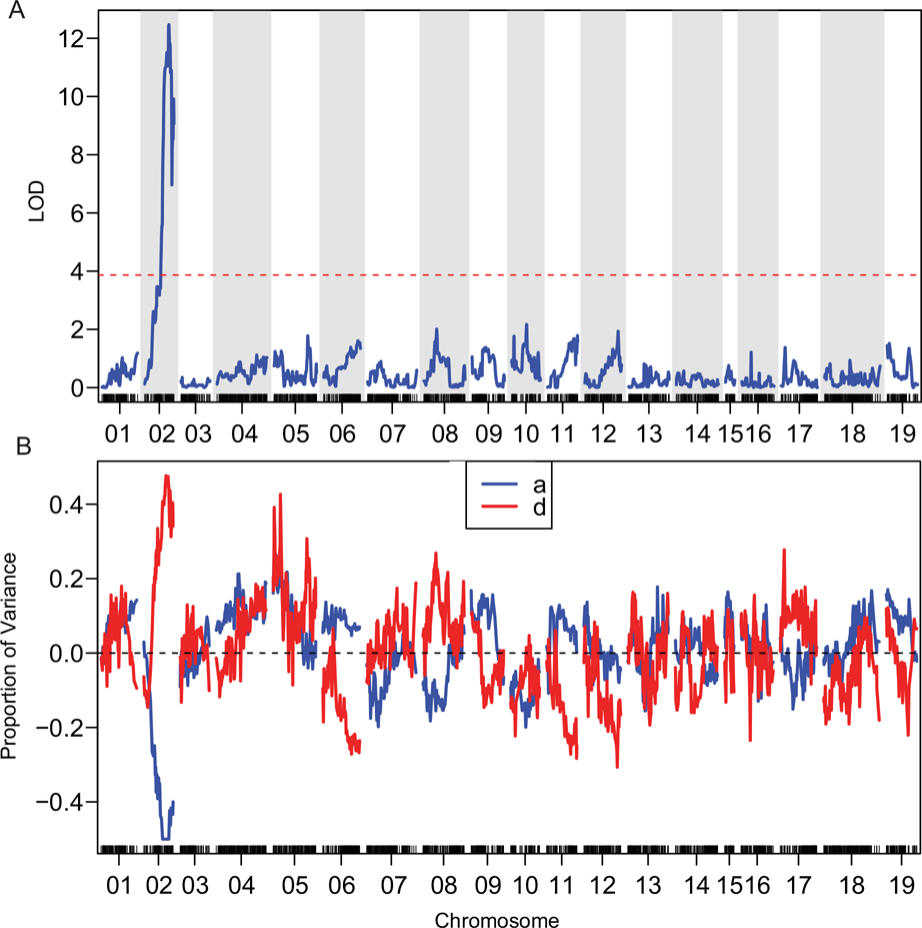
Berry color segregation used for genetic map validation. (A) QTL for berry skin color is confirmed on Chr02 of the genetic map. Permutation tests were carried out to identify the 95% confidence threshold, and the significance threshold for the LOD score is presented as a horizontal red dashed line. (B) The additive effect (a, blue line) and dominant deviation (d, red line) are estimated across the whole genome.

Berry skin color is controlled by the *VvmybA1* gene (Chr02: 14,179,266–14,180,746) [58]. This well-studied trait was used to validate the soundness of the F_2_ map construction strategy. Berry skin color was recorded for 65 F_2_ progeny and showed a 51:14 ratio of black:white (pigmented:nonpigmented), which was not significantly different from 3:1 (Chi-squared test, p = 0.80, S4 File), confirming the trait was controlled by one completely-dominant gene. Only one significant QTL, with a peak LOD score of 12.47 (threshold = 3.9), was identified on LG02, and explained up to 90% of the phenotypic variance for berry skin color (Fig 3A). The 1.5-LOD support interval spanned the genetic map at 71.06–90.85cM, corresponding to Chr02 6.3–14.3Mbp and encompassed the *VvmybA1* locus (Table 2). The estimated additive effect was 0.5 (a = 0.50), equal to the estimated dominant deviation (d = 0.46), which perfectly matched the one-locus, complete-dominance model and thereby validated the F_2_ map construction strategy (Fig 3B).

**Table 2:** Summary of QTL analyses in *V*. *riparia* × ‘Seyval’ F_2_ family.

Trait	No. of individuals phenotyped	Linkage Group (LG)	Peak position (cM) ^a^	Physical position of the nearest marker	LOD score	LOD interval (cM) ^b^	LOD interval (physical location)	Additive effect ^c^	Dominant effect	R^2^ ^d^
Berry skin color	65	2	85.40	9,126,568	12.47	71.06–90.85	6,360,259–14,300,736	-0.50	0.47	89.92
Yeast assimilable nitrogen (YAN, mg/L)	63	7	100.44	18,819,915	6.48	94.87–106.85	16,987,038–20,565,550	-52.55	-38.91	22.81
Malic acid concentration (MA, g/L)	63	6	70.24	7,985,435	6.24	62.95–75.23	7,343,698–8,358,059	2.50	1.26	26.19
Total soluble solids content (SS, %w/w)	63	6	46.18	5,498,696	4.76	35.85–54.71	4,258,443–6,497,323	-2.16	0.0811	19.14
MA/SS ratio	63	6	32.10	3,857,140	5.93	27.07–35.85	3,524,817–4,258,443	0.17	0.00262	26.04
Joint analysis of MA and SS	63	1	49.94	5,061,702	6.55	49.10–51.12	5,026,255–5,123,842	MA -0.40	0.34	7.93
								SS -0.14	0.20	1.02
Joint analysis of MA and SS	63	6	75.23	8,358,059	4.23	70.21–80.83	7,985,435–11,876,418	MA 0.60	0.28	18.37
								SS 0.62	-0.24	19.43

^**a**^ Position of likelihood peak (highest LOD score).

^**b**^ LOD interval refers to 1.8-LOD support interval, except for Berry skin color, for which a 1.5-LOD support interval was used.

^**c**^ Additive effect: A positive value means the higher value of the trait due to allele from grandparent2, ‘Seyval’. A negative value means the higher value of the traits due to allele from grandparent1, *V*. *riparia*. The units depend on the traits.

^**d**^ R^2^ (coefficient of determination): percentage of phenotypic variance explained by the QTL.

### QTL mapping for enological traits

**Fig 4:**
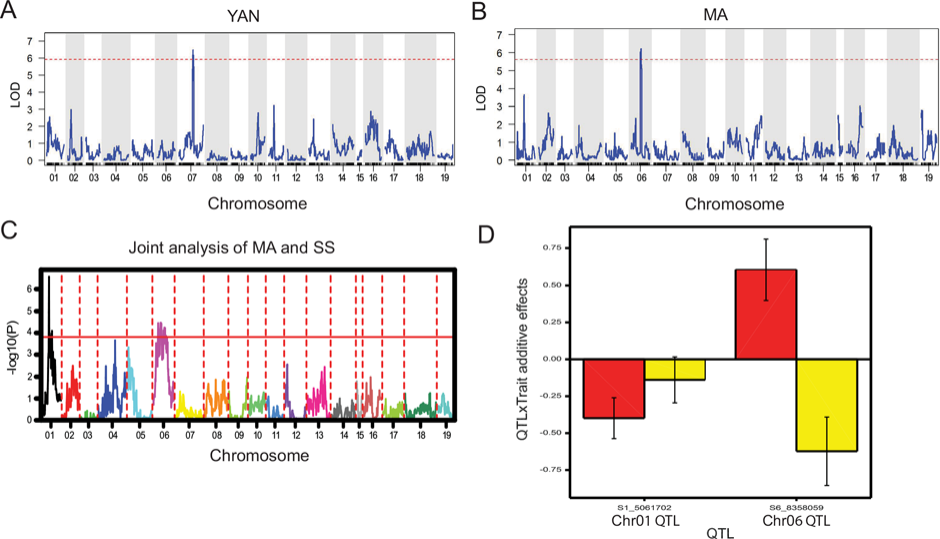
QTL mapping for quantitative enological traits. (A) QTL mapping of yeast assimilable nitrogen (YAN, mg/L). (B) QTL mapping of malic acid concentration (MA, g/L). (C) The joint QTL mapping of MA (g/L) and total soluble solids content (SS, %w/w). Permutation tests were carried out to identify the 95% confidence threshold, and the significance thresholds of LOD score is presented as a horizontal red line in A-C. (D) The interaction between MA (g/L) and SS (%w/w) QTLs and trait additive effects. Red bars represent additive effects of MA and yellow bars represent additive effects of SS. A positive value means the higher value of the trait is due to an allele from grandparent 2, ‘Seyval’. A negative value means the higher value of the trait is due to an allele from grandparent 1, *V*. *riparia*.

QTL mapping of the enological traits YAN, MA and SS was conducted using composite interval mapping (CIM) and the Kosambi mapping function. Each phenotype followed a normal distribution (S4 File). A QTL corresponding to YAN was identified on LG07 with a peak at 100.44 cM (LOD = 6.48; Fig 4A). This YAN QTL spanned a physical interval from 17.0–20.6 Mbp (1.8-LOD support interval) and explained 22.8% of phenotypic variance (Table 2). The additive effect of the QTL was negative, indicating that higher concentrations of YAN came from the grandparent 1, *V*. *riparia*. For MA, a single QTL was observed on LG06 with a peak at 70.24 cM (LOD = 6.24). This MA QTL contained a relatively narrow 1.8-LOD support interval from 7.3–8.4 Mbp and explained 26.2% of the phenotypic variance (Fig 4B, Table 2). In contrast to YAN, the positive additive effect of MA refers to higher concentration in the grandparent 2, ‘Seyval’. For the ratio of MA/SS, a single QTL was identified on LG06, peaking at 32.10 cM (LOD = 5.93). This MA/SS QTL spanned a relatively narrow interval between 3.5 and 4.3 Mbp and explained 26.0% of the phenotypic variance (Table 2). The additive effect was positive, indicating that higher MA/SS ratios came from ‘Seyval’, and the dominant effect was negligible.

Since the QTL analysis was performed on data for a single year and single location, data transformation and multiple QTL detection methods and software programs were applied to confirm the results. The joint analysis of SS and MA showed evidence of two QTLs affecting both traits (Fig 4C). One QTL was detected at LG01 at the 49.94cM (5.0 Mbp), for which the additive effects indicated that the traits came from the grandparent 1, *V*. *riparia* (Table 2). This QTL explained around 7.9% and 1.0% of the phenotypic variance for MA and SS, respectively. Another QTL on LG06 at 75.23cM (8.3 Mbp) also showed high evidence for influencing both traits simultaneously; however, the additive positive effect on MA came from grandparent 2, ‘Seyval’, and the additive effect on SS came from *V*. *riparia*. This particular QTL was noteworthy since it explained around 18.3 and 19.4% of the phenotypic variance of MA and SS, respectively, and co-localized with the MA QTL identified in the CIM analysis. No significant dominance effect was observed for either trait. The interaction between QTL and trait additive effects was significant (Fig 4D). Thus for SS, both QTLs showed negative additive effects (-0.14 and -0.62, for LG01 and LG06, respectively), while for MA, the QTL on LG01 showed a negative effect and the QTL on LG06 positive (-0.40 and 0.60, respectively).

The results from the causal model selection hypothesis tests showed that the values for *Bayesian Information Criterion* (BIC) and *Akaike Information Criterion* (AIC) did not agree in their most adequate model (S5 File). The AIC value suggested that the full model with nine parameters was best, and that the traits were affected by more than genetic factors. The BIC value suggested that considering SS as phenotype 1, the best model was a causal effect of SS on MA. When MA was considered as phenotype 1, the BIC value indicated the reactive model was best, confirming that MA was reacting to a causal effect of SS.

## Discussion

Over the last ten years, linkage map construction has been widely used in grapevine genetic research, with markers primarily being amplified fragment length polymorphisms (AFLPs), SSRs and SNPs [27]. Although SSRs have been widely used in grapevine linkage map construction, the method is relatively time consuming and costly, typically resulting in low marker density. For example, 119 individuals from the mapping family used here were previously genotyped using 115 SSRs [29], which was much less than that required to capture all the informative recombination. That SSR map was unable to cover the whole genome and resulted in six gaps larger than 20 cM due to uneven distribution, which would result in information loss if causal alleles were located in the missing region.

In contrast, GBS-based SNPs can provide high-density genetic maps and are conducive to high-throughput genotyping. The genetic map for this F2 population (Fig 2, Table 1) consisted of 1,449 markers 12.6-fold increase in marker density compared to the earlier SSR map. The maximum spacing between markers was reduced to 13.5 cM, and 15 out of 19 chromosomes have maximum gaps less than 10 cM, resulting in an average distance of 1.67 cM/marker. The GBS markers are almost evenly distributed across the genome, and cover 95% of the ‘PN40024’ reference genome. This high-resolution genetic map should better detect recombination events. Since linkage disequilibrium (LD) is low in *Vitis* due to its heterozygosity and diversity [11, 59], greater marker density increases the odds of finding markers for specific traits, or even to fine map candidate causal genes. Further, the GBS protocol is well established, publicly available, and can be scaled efficiently up to the current 384-plex barcoding system [35], at which the total genotyping cost is currently less than $15 per sample. Thus, GBS-SNP markers are a time- and cost-efficient method for high resolution genetic map construction and potentially for marker-assisted selection. However, current GBS pipelines have been designed for inbred germplasm with very low residual heterozygosity and do not provide methodology for obtaining intercross markers, for which heterozygous materials are enriched and for which the marker phase is not known.

The co-dominant intercross markers of an F_2_ mapping family enabled the capture of additional meiotic events relative to the F_1_ pseudo-testcross mapping approach. Furthermore, in an F_1_ family, only QTLs in heterozygous allelic states in the parents with large allele-effect differentials can be detected [60], which complicates the estimation of additive effects contributed by each parent. Thus, F_2_ families with at least 200 progeny genotyped with intercross markers have superior linkage map accuracy [61, 62] and allow the estimation of additive, and to some extent dominant, genetic effects contributed per parent [63]. There are two types of markers in an F_2_ mapping family: 1:2:1 segregation of co-dominant markers, which are more informative than 3:1 segregation of dominant markers.

Here, we addressed several specific challenges associated with GBS analysis, particularly of a heterozygous family. First, retrieving useful GBS markers for map generation poses a challenge, since there is still a gap between the standard GBS variant calling pipeline TASSEL and commercial map construction software JoinMap. Also, heterozygous genotypes may be called due to sequencing errors or genome structure variance such as paralogs or tandem duplication. When imputation is difficult to apply on highly heterozygous species, pre-filtering and marker type identification and selection need to be carefully performed. The custom perl script (S1 File) provided in this paper identified markers with a co-dominant segregation (i.e. 1:2:1) in the progeny and verified each marker against the allelic state of the progenitor. To our knowledge, this is the first publication using intercross SNP markers for map construction in an F_2_ progeny in grapevine. The perl script can be used for marker development in other highly heterozygous species, such as apple, poplar and willow.

To validate the marker development and map construction strategy, the trait of berry color was subjected to QTL analysis (Fig 3). Berry color was selected for map validation for two reasons: (1) it is a highly heritable binary trait that is easy to phenotype; and (2) the genetic basis is well studied. Specifically, the *VvmybA1* gene (Chr02: 14,179,266–14,180,746) regulates anthocyanin pigment biosynthesis in grapes [64], and a retrotransposon-induced mutation in *VvmybA1* has been identified as the molecular basis of white cultivars of *V*. *vinifera* [58]. The QTL detected at 14.2 Mbp on Chr02 aligned perfectly with *VvmybA1*, indicating our approach was effective, even with a small sample size of 65 phenotyped progeny. The GBS genetic map was also compared to a physical map based on the reference genome *V*. *vinifera* ‘PN40024’ (S3 File). The majority of co-linearity indicated that marker order is well conserved even across the genome segments of diverse *Vitis* species represented in the parents, including *V*. *vinifera*, *V*. *rupestris*, *V*. *aestivalis* var *lincecumii* and *V*. *riparia*. These markers can be used as anchors to improve the ‘PN40024’ reference genome assembly by filling existing gaps or incorporating random contigs. However, there are some chromosomal regions that show weaker correlation between genetic and physical maps, e.g. the upper end of Chr16. These regions might indicate species-specific genome structure variation, such as chromosome rearrangement, transposable elements and tandem duplication. *V*. *vinifera* is the only species in the *Vitis* complex with a whole genome sequence already published, and the *de novo* genome assembly for other *Vitis* species can provide insight into unique characteristics arising from adaption to local conditions. Thus, this interspecific genetic map can be viewed as an early step toward a grapevine pan-genome, as pursued in maize [64].

Once the genetic map was generated and validated, three berry traits related to winegrape quality were chosen for QTL analysis. YAN–or the sum of nitrogen contained in primary amino acids and ammonium–is the nitrogenous component of grapes that can be utilized by yeast during alcoholic fermentation. Proper nitrogen concentrations are of interest to winemakers since nitrogen deficiency is associated with sluggish or stuck fermentations, increased production of off-aroma compounds like H_2_S, and decreased formation of desirable odorants like esters [1, 65]. Conversely, excess YAN can cause several problems, most notably formation of carcinogenic ethyl carbamate. Typically, ammonium is accumulated first, and decreases during ripening with corresponding increases in primary amino nitrogen [1]. Total YAN changes relatively little during berry ripening–across a wide range of cultivars and sites, YAN concentrations at commercial harvest averaged only 25% higher and were well correlated with values measured up to 5 weeks prior [66]. However, genotype can have a profound effect on YAN–up to a three-fold variation among *vinifera* cultivars (75 mg/L vs. 220 mg/L) was observed in a survey of cultivars in one growing region [66], and much larger variations were observed among interspecific hybrids [18]. We observed a YAN range in our mapping family (100–600 mg/L) greater than this previous survey, further confirming the importance of genetics in YAN.

Currently there is little information on the genetics of YAN and this, to our knowledge, is the first YAN associated QTL in grapevine. The QTL results in this study revealed one locus associated with YAN on LG07 (LOD = 6.48) from 16,987,038 to 20,565,550 bp, within the 1.8-LOD support interval (Table 2). Thirteen genes relevant to nitrogen metabolism were identified within the interval and can be used to further explore the trait. Because berries appear to accumulate ammonium, which is subsequently metabolized to primary amino acids, the presence of ammonium transporter genes in this list (*AMT2*) is of particular interest. The altered function of these genes could limit either N uptake by the vines or YAN accumulation in the berries. The estimated additive effect of the QTL indicated the *V*. *riparia* grandparent contained a higher concentration of YAN than ‘Seyval’ and may provide a novel tool for research into the nature of nitrogen content in grape berries.

Both MA and SS are critical parameters for evaluating fruit maturity, where the primary component of SS in mature fruit is hexose sugars (fructose, glucose). The key biochemical pathways associated with MA and hexose sugar metabolism are well-established [67]. Pre-veraison, sucrose imported into grape berries via the phloem is used as the primary metabolic substrate. Concurrently, a portion of malate from the citric acid cycle is transported and stored in vacuoles, where it serves as a key contributor (along with tartaric acid) to the high TA of unripe grapes [68]. During veraison and ripening, grapes accumulate sugars, and shift to using MA as an energy source [14]. Thus, a key change associated with veraison is an increase in SS (caused primarily by inversion of imported sucrose and accumulation of resulting hexose sugars) coinciding with a reduction in TA (caused primarily because of MA respiration). Grape crosses with non-domesticated *Vitis* in their background, e.g. *V*. *riparia*, can have unacceptably high TA values and MA concentrations (>10 g/L) even at normal harvest SS values (>20 Brix) [5]. The family used in this study shows this problem with over 50% of individuals having MA > 10 g/L (S4 File).

Because MA and SS are co-regulated, QTL analyses of individual traits were complemented by two other approaches 1) QTL analysis of the ratio, MA/SS, and 2) joint (simultaneous multi-trait) analysis of MA and SS. Both techniques have advantages over the trait-by-trait analysis, the most relevant being the increase in power to identify and accurately locate QTLs [69–72]. However, both approaches also provide additional information that is not easily obtained when traits are analyzed separately, such as the power to identify loci involved in the regulation at specific metabolic branch-points and infer interaction among genetic components for the exhibition of the traits.

The analysis of two traits as a ratio creates some difficulties, because when two traits are identically normally distributed, their ratio follows a Cauchy distribution as opposed to the normal distribution expected by most algorithms [71]. However, the mapping of these ratio-traits is relevant because it allows researchers to test and infer the interaction between traits, exemplified here by the interaction of compounds that have relevance for harvest decision-making in the field and fermentation must manipulations in the winery. From a genetics perspective, the analysis of ratio-traits allows the potential opportunity to integrate and discover independent, closely-linked, and pleiotropic loci [73] when the separated traits exhibit a significant positive or negative correlation. For example, MA/SS ratio would be less sensitive than the individual traits to changes in berry size.

In our study, the analysis of the MA/SS ratio showed a QTL on LG06 distal from the QTLs identified for SS and MA when analyzed separately (Table 2, S6 File). The LOD-based evidence (4.77–6.24) and amount of variance explained (19–26%) was comparable for the traits analyzed individually or as a ratio. The interpretation of the additive and dominance effects suggested that the higher value for the ratio could be due to the genetic contribution from ‘Seyval’; however, since this ratio can be interpreted as a biochemical interaction, the interpretation may require additional inference to suggest what is being genetically inherited.

The joint analysis of multiple traits includes all traits of interest simultaneously in a single model. This approach can provide insights into fundamental genetic mechanisms underlying trait relationships such as pleiotropy and close linkage, genotype-by-environment (G × E) interactions, and the possible trade-off given for negatively correlated traits [70]. This joint analysis approach generated a list of 14 candidate genes within the QTL region. The joint analysis showed a QTL detected on Chr06 with 1.8-LOD support interval from 70.2–80.8 cM (Table 2). The corresponding physical interval, Chr06:7,985,435–11,876,418, covered the region of grapevine orthologs of the aluminum activated malate transporter, ALMT1. This gene corresponds to the *Ma1* and *Ma2* loci in apple, which are malic acid transporters controlling the acidity level in apple fruits [74]. Our observation aligns with a QTL for MA at 74.1 cM on Chr06, reported by another group using a *Vitis* spp. mapping family [20]. The QTL explained 17% of the variance for MA in one year of a three year study. This group did not implicate ALMT1 as a candidate gene in Chr06, although they did observe a more minor QTL on Chr18, which they suggested could be related to a different ALMT. Interestingly, significant QTL were not detected for other genes expected to control MA concentrations. For example, MA respiration rate, the rate at which grape berries metabolize MA after veraison, is dependent on NAD dependent malate enzyme [68], but no QTL was detected at its genetic location. QTL analyses of MA are complicated because high MA at harvest could arise from either high MA accumulation pre-veraison or slow MA respiration, and future studies could likely benefit from having multiple time points, i.e. pre- and post-veraison.

The analysis of multiple traits simultaneously implies advantages when causal relationships between the studied traits and genes or among traits exist; however, when the trait is affected by a gene through a transitional trait, the advantages almost disappear [72]. For such a reason the testing of the causal relationships (correlation, pleiotropy, or close linkage) between or among traits must be addressed to yield outputs that can be interpreted biologically. Also, the addition of more traits to the joint QTL analysis does not result in more detection power, but may give rise to spurious signals since additional parameters will be fitted in the model [75]. However, to break unfavorable linkages of genes involved in the exhibition of economically important traits, the separation of closely linked loci is needed to eliminate pleiotropic negative effects [75].

In this study, the test for causal relationships between SS and MA was pursued through causal model selection hypothesis tests [56]. The results from CMST showed that a causal relationship clearly exists between MA and SS with a common genetic component (QTL), as the model for independence was not significant in any of the tests. AIC values were minimized for the full model, indicating SS and MA share more than one common genetic component. While, BIC values were minimized for models showing a casual effect of a QTL controlling SS also affecting MA. The discrepancy between AIC and BIC is not a concern for the interpretation of the results in the light of the empirical experience for these traits. Therefore, the results are compatible with the empirical experience, in which the balance between MA and SS is intertwined and influenced by several other factors, such as environment (particularly temperature), field management (irrigation, fertilization), and biology (additional biochemical interactions). Hence, genetic research, breeding, and probably biochemistry approaches, should consider MA and SS simultaneously, as well as the possible interactions with non-genetic factors.

## Conclusions

The results presented here confirmed the GBS mapping strategy for an expanded F_2_ mapping family in grapevine. The protocol presented here filters heterozygous markers and retrieves high quality intercross markers which are phased during linkage map development and allows QTL analysis using an F_2_ genetic design, which is not common for grapevine genetic research. In addition, the protocols for calling intercross GBS markers could be used for other heterozygous species. The linkage map provided qualitative and quantitative improvements over a previous SSR map for marker density and genome coverage while maintaining co-linearity with the reference genome. The availability of the linkage map facilitated the genetic study of winegrape quality traits, including a well-characterized binary trait (berry color), more complicated traits (malic acid, soluble solids) and a neglected trait (yeast assimilable nitrogen). Enhanced marker resolution provided the opportunity to conduct joint analysis of malic acid and soluble solids and results indicated these compounds are intertwined and should be considered jointly in future genetic studies. The resources generated here can further guide the study and manipulation of fruit harvest, must processing and wine fermentation, and may also contribute to decision making for breeding and cultivar development of grapevines. The novel GBS analysis methods presented here could have broad impact for F_2_ mapping in diverse heterozygous species.

## Supporting Information

S1 File Perl code for obtaining intercross markers. Perl code to identify markers from TASSEL hapmap file that are (1) heterozygous in the F_1_ progenitor and (2) show co-dominant 1:2:1 segregation among the F_2_ progeny by a chi-squared (χ^2^) goodness-of-fit test at α≤0.01.
(PL) Click here for additional data file.

S2 File GBS-based linkage map statistics. Summary statistics of the *V*. *riparia* × ‘Seyval’ F_2_ GBS-based linkage map.
(DOCX)

S3 File Comparison between GBS genetic map and physical map. X-axes represent physical coordinates in the reference genome *V*. *vinifera* ‘PN40024’ version 12X.v2 (Mb). Y-axes represent genetic coordinates (cM).
(PDF)

S4 File Distribution of traits in the *V*. *riparia* × ‘Seyval’ F_2_ family. (A) Sixty five F_2_ progeny were measured for berry skin color. White (nonpigmented) is coded as 0 and black (pigmented) is coded as 1. (B-D) Distribution frequency for quantitative enological traits in 63 F_2_ progeny.
(PDF)

S5 File Results from the causal model selection test. Log-likelihood (LogLik), Akaike Information Criterion (AIC) and Bayesian Information Criterion (BIC) evaluated for each of the causal models considered in CMST. In the column “Test”, a simple directed acyclic diagram is shown as a representation of the test performed. In this particular case, a genetic component-QTL (Q) influences either of the traits (MA or SS). The arrows represent the direction of the influence, which can go from Q to either trait or from trait to trait. Influence of trait upon Q is not considered in this approach. The values associated with the most likely models are highlighted in bold.
(DOCX)

S6 File QTL mapping for berry quality traits. QTL mapping for (A) total soluble solids content (SS, %w/w) and (B) the ratio of malic acid concentration (MA, g/L) to total soluble solids content (SS, %w/w). Permutation tests were carried out to identify 95% confidence thresholds, and the significance threshold of LOD score is presented as a horizontal red dashed line.
(PDF)

S7 File SNP marker sequences. The sequence of markers in the final map.
(FASTA)
