# Supplementary material for: Next Generation Mapping of Enological Traits in an F2 Interspecific Grapevine Hybrid Family
Source: PLoS One. 2016 Mar 14;11(3):e0149560. doi: 10.1371/journal.pone.0149560 (PMC4790954; doi:10.1371/journal.pone.0149560)
Supplement: S2 File — Summary statistics of the V. riparia × ‘Seyval’ F2 GBS-based linkage map. (DOCX) [file pone.0149560.s002.docx]

| Linkage Group | No. of markers | Genetic Length (cM) | Ave Spacing (cM) ^a^ | Max Spacing (cM) ^b^ | Physical Length (bp) ^c^ | Coverage (%) ^d^ |
| --- | --- | --- | --- | --- | --- | --- |
| 1 | 70 | 125.4 | 1.8 | 9.5 | 24233538 | 90.4 |
| 2 | 59 | 103.0 | 1.8 | 8.0 | 18891843 | 98.5 |
| 3 | 61 | 102.2 | 1.7 | 8.0 | 20695524 | 92.5 |
| 4 | 114 | 175.6 | 1.6 | 6.3 | 24711646 | 97.6 |
| 5 | 100 | 149.8 | 1.5 | 5.2 | 25650743 | 99.2 |
| 6 | 80 | 129.7 | 1.6 | 6.7 | 22645733 | 97.2 |
| 7 | 97 | 173.4 | 1.8 | 8.4 | 27355740 | 98.4 |
| 8 | 89 | 144.8 | 1.6 | 5.9 | 22550362 | 98.2 |
| 9 | 73 | 114.0 | 1.6 | 10.7 | 23006712 | 98.4 |
| 10 | 49 | 101.6 | 2.1 | 13.5 | 23503040 | 96.2 |
| 11 | 56 | 106.5 | 1.9 | 7.6 | 20118820 | 97.6 |
| 12 | 73 | 129.4 | 1.8 | 5.2 | 24269032 | 99.0 |
| 13 | 93 | 143.1 | 1.6 | 7.9 | 29075116 | 99.1 |
| 14 | 93 | 148.4 | 1.6 | 6.3 | 30274277 | 98.4 |
| 15 | 21 | 32.6 | 1.6 | 3.6 | 20304914 | 61.3 |
| 16 | 68 | 113.3 | 1.7 | 6.8 | 23572818 | 96.4 |
| 17 | 81 | 128.2 | 1.6 | 8.9 | 18691847 | 91.4 |
| 18 | 116 | 195.4 | 1.7 | 10.5 | 34568450 | 98.5 |
| 19 | 56 | 107.3 | 2.0 | 12.2 | 24695667 | 98.3 |
| overall | 1449 | 2423.9 | 1.7 | 13.5 | 458815822 | 95.1 |

**S2 File.** Summary statistics of *V. riparia* × ‘Seyval’ **F_2_ GBS-based linkage map**.

^a^ Ave Spacing (cM) refers to the average genetic distance between two adjacent markers in each chromosome.

^b^ Max Spacing (cM) refers to the maximum genetic distance between two adjacent markers in each chromosome.

^c^ Physical Length (bp) is chromosome length of 12x.2 *V. vinifera* ‘PN40024’ reference genome covered by this linkage map.

^d^ Coverage (%) is calculated as the Physical Length presented for this linkage map divided by the total physical length of the chromosome in the ‘PN40024’ reference genome.
