# Supplementary figures and images for: Next Generation Mapping of Enological Traits in an F2 Interspecific Grapevine Hybrid Family
Source: PLoS One. 2016 Mar 14;11(3):e0149560. doi: 10.1371/journal.pone.0149560 (PMC4790954; doi:10.1371/journal.pone.0149560)

Chr 1

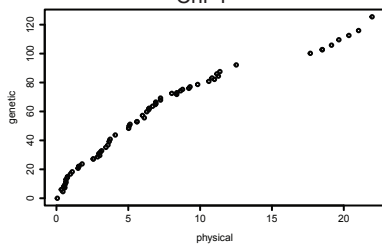

Chr 2

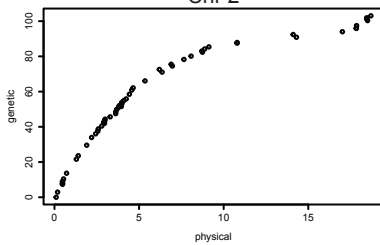

Chr 3

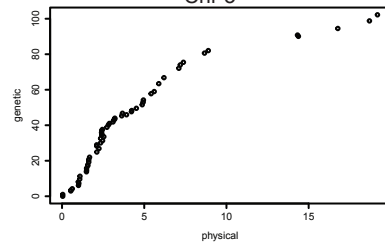

Chr 4

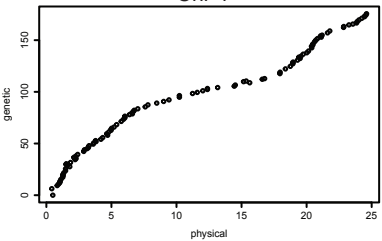

Chr 5

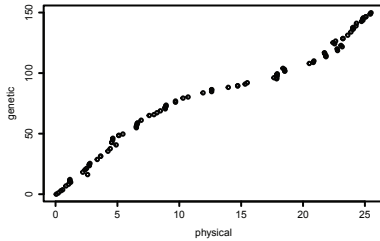

Chr 6

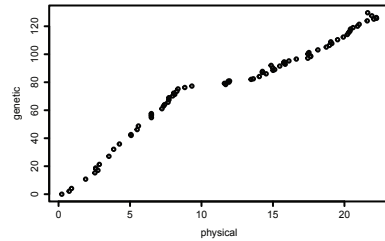

Chr 7

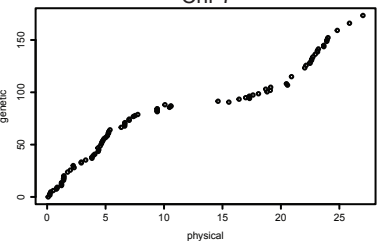

Chr 8

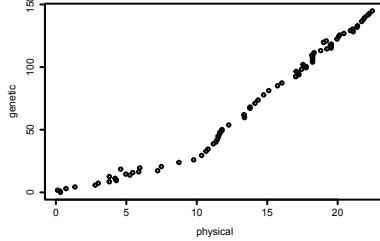

Chr 9

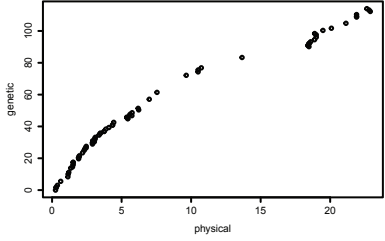

Chr 10

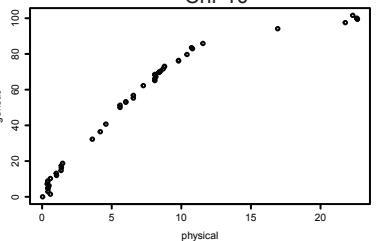

Chr 11

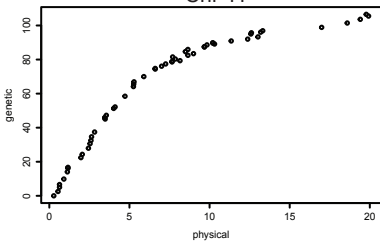

Chr 12

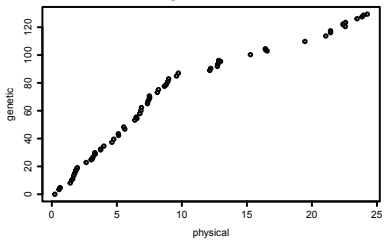

Chr 13

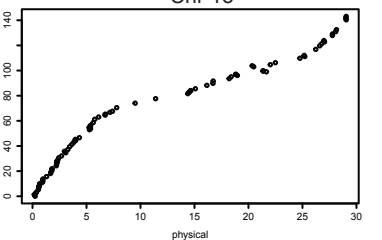

Chr 14

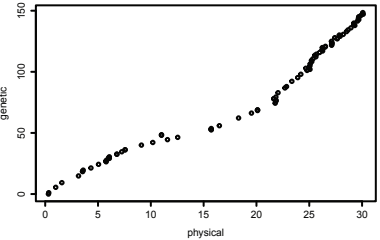

Chr 15

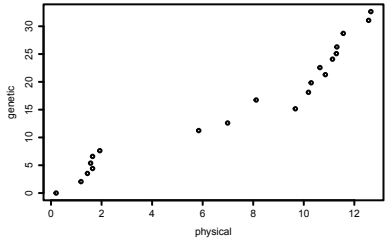

Chr 16

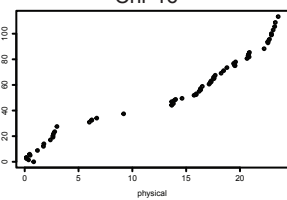

Chr 17

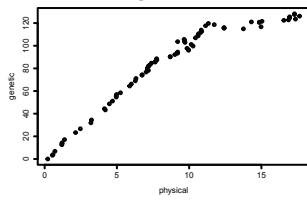

Chr 18

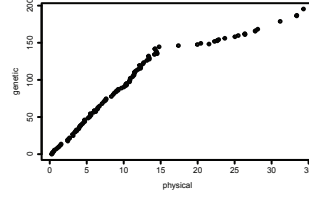

Chr 19

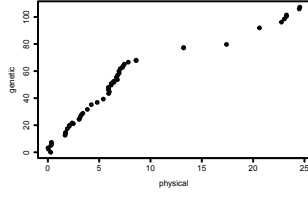

Supplement: S3 File — X-axes represent physical coordinates in the reference genome V. vinifera ‘PN40024’ version 12X.v2 (Mb). Y-axes represent genetic coordinates (cM). (PDF) [file pone.0149560.s003.pdf]

A

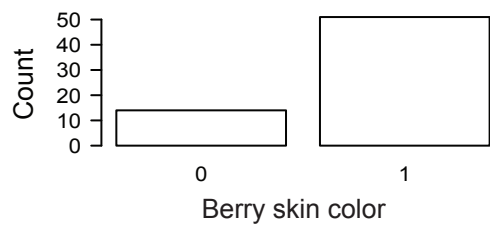

B

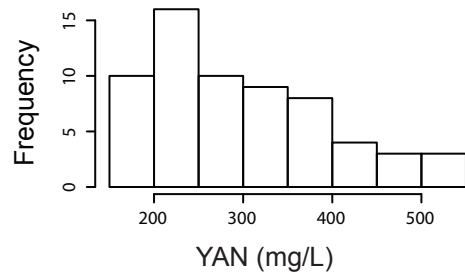

C

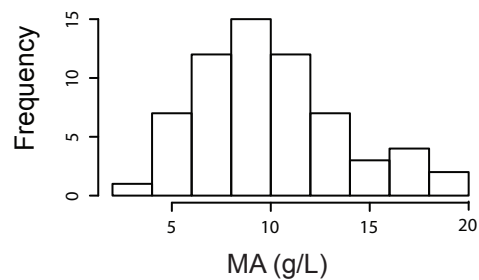

D

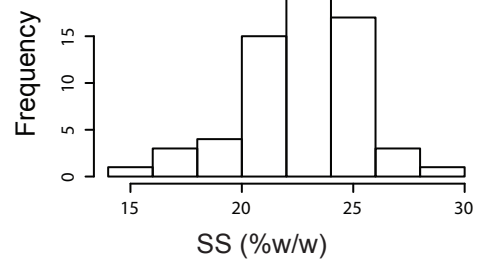

E

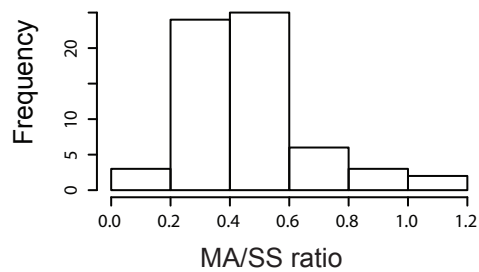

Supplement: S4 File — (A) Sixty five F2 progeny were measured for berry skin color. White (nonpigmented) is coded as 0 and black (pigmented) is coded as 1. (B-D) Distribution frequency for quantitative enological traits in 63 F2 progeny. (PDF) [file pone.0149560.s004.pdf]

**A****SS**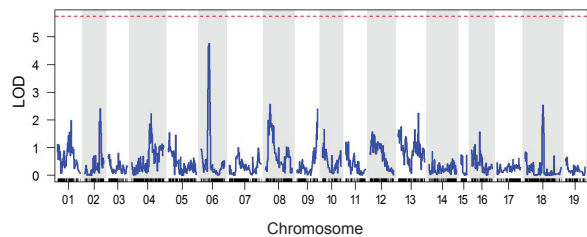**B****MA/SS ratio**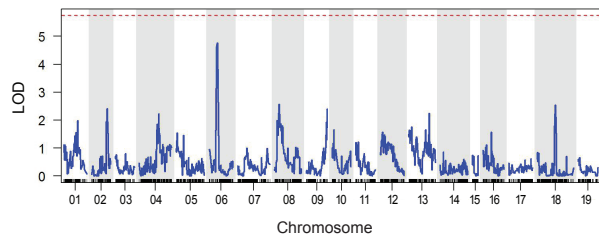

Supplement: S6 File — QTL mapping for (A) total soluble solids content (SS, %w/w) and (B) the ratio of malic acid concentration (MA, g/L) to total soluble solids content (SS, %w/w). Permutation tests were carried out to identify 95% confidence thresholds, and the significance threshold of LOD score is presented as a horizontal red dashed line. (PDF) [file pone.0149560.s006.pdf]
