# Supplementary material for: Next Generation Mapping of Enological Traits in an F2 Interspecific Grapevine Hybrid Family
Source: PLoS One. 2016 Mar 14;11(3):e0149560. doi: 10.1371/journal.pone.0149560 (PMC4790954; doi:10.1371/journal.pone.0149560)
Supplement: S5 File — Log-likelihood (LogLik), Akaike Information Criterion (AIC) and Bayesian Information Criterion (BIC) evaluated for each of the causal models considered in CMST. In the column “Test”, a simple directed acyclic diagram is shown as a representation of the test performed. In this particular case, a genetic component-QTL (Q) influences either of the traits (MA or SS). The arrows represent the direction of the influence, which can go from Q to either trait or from trait to trait. Influence of trait upon Q is not considered in this approach. The values associated with the most likely models are highlighted in bold. (DOCX) [file pone.0149560.s005.docx]

**S5 File. Results from the** causal model selection test. Log-likelihood (LogLik), Akaike Information Criterion (AIC) and Bayesian Information Criterion (BIC) evaluated for each of the causal models considered in CMST. In the column “Test”, a simple directed acyclic diagram is shown as a representation of the test performed. In this particular case, a genetic component-QTL (Q) influences either of the traits (MA or SS). The arrows represent the direction of the influence, which can go from Q to either trait or from trait to trait. Influence of trait upon Q is not considered in this approach. The values associated with the most likely models are highlighted in bold.

| Model | Test | LogLik | AIC | BIC |
| --- | --- | --- | --- | --- |
| *M1*=Casual effect  (7 parameters) | $\begin{matrix} & Q & \\ ↙ & & \\ MA & \to& SS \end{matrix}$ | -306.71 | 627.43 | 642.43 |
|  | $\begin{matrix} & Q & \\ ↙ & & \\ SS & \to& MA \end{matrix}$ | -305.10 | 624.21 | **639.21** |
| *M2* = Reactive effect  (7 parameters) | $\begin{matrix} & Q & \\ & & ↘ \\ MA & \leftarrow& SS \end{matrix}$ | -305.10 | 624.21 | **639.21** |
|  | $\begin{matrix} & Q & \\ & & ↘ \\ SS & \leftarrow& MA \end{matrix}$ | -306.71 | 627.43 | 642.43 |
| *M3* = Correlation due to common QTL  (8 parameters) | $\begin{matrix} & Q & \\ ↙ & & ↘ \\ MA & & SS \end{matrix}$ | -307.31 | 630.62 | 647.76 |
|  | $\begin{matrix} & Q & \\ ↙ & & ↘ \\ SS & & MA \end{matrix}$ | -307.31 | 630.62 | 647.76 |
| *M4* = Full  (9 parameters) | $\begin{matrix} & Q & \\ ↙ & & ↘ \\ MA & \leftrightarrow& SS \end{matrix}$ | -302.41 | **622.81** | 642.10 |
|  | $\begin{matrix} & Q & \\ ↙ & & ↘ \\ SS & \leftrightarrow& MA \end{matrix}$ | -302.41 | **622.81** | 642.10 |
